# Leaving No Child Behind: Decomposing Socioeconomic Inequalities in Child Health for India and South Africa

**DOI:** 10.3390/ijerph18137114

**Published:** 2021-07-02

**Authors:** Olufunke A. Alaba, Charles Hongoro, Aquina Thulare, Akim Tafadzwa Lukwa

**Affiliations:** 1 Health Economics Unit, School of Public Health and Family Medicine, Faculty of Health Sciences, University of Cape Town, Anzio Road, Observatory, Cape Town 7925, South Africa; tafadzwalukwa@gmail.com; 2 Developmental, Capable and Ethical State, Human Sciences Research Council, Pretoria 0001, South Africa; chongoro@hsrc.ac.za; 3 School of Health Systems and Public Health (SHSPH), Faculty of Health Sciences, University of Pretoria, Pretoria 0028, South Africa; 4 National Department of Health, Pretoria 0001, South Africa; aquina.thulare@gmail.com

**Keywords:** Universal Health Coverage, decomposing socioeconomic inequalities, food insecurity, immunization, malnutrition, child health, under-five

## Abstract

Background: The United Nations’ 2030 Agenda for Sustainable Development argues for the combating of health inequalities within and among countries, advocating for “leaving no one behind”. However, child mortality in developing countries is still high and mainly driven by lack of immunization, food insecurity and nutritional deficiency. The confounding problem is the existence of socioeconomic inequalities among the richest and poorest. Thus, comparing South Africa’s and India’s Demographic and Health Surveys (DHS) of 2015/16, this study examines socioeconomic inequalities in under-five children’s health and its associated factors using three child health indications: full immunization coverage, food insecurity and malnutrition. Methods: Erreygers Normalized concentration indices were computed to show how immunization coverage, food insecurity and malnutrition in children varied across socioeconomic groups (household wealth). Concentration curves were plotted to show the cumulative share of immunization coverage, food insecurity and malnutrition against the cumulative share of children ranked from poorest to richest. Subsequent decomposition analysis identified vital factors underpinning the observed socioeconomic inequalities. Results: The results confirm a strong socioeconomic gradient in food security and malnutrition in India and South Africa. However, while full childhood immunization in South Africa was pro-poor (−0.0236), in India, it was pro-rich (0.1640). Decomposed results reported socioeconomic status, residence, mother’s education, and mother’s age as primary drivers of health inequalities in full immunization, food security and nutrition among children in both countries. Conclusions: The main drivers of the socioeconomic inequalities in both countries across the child health outcomes (full immunization, food insecurity and malnutrition) are socioeconomic status, residence, mother’s education, and mother’s age. In conclusion, if socioeconomic inequalities in children’s health especially food insecurity and malnutrition in South Africa; food insecurity, malnutrition and immunization in India are not addressed then definitely “some under-five children will be left behind”.

## 1. Introduction

As of 2018, about 6.2 million children died from preventable causes; however, out of these deaths, 5.3 million occurred in the first five years and almost half occurred in the first month of life [1]. However, more than half of these early child deaths were preventable by simple, affordable interventions, including immunization, adequate nutrition, safe water and food [2,3,4,5,6,7]. Children in sub-Saharan Africa are 15 times more likely to die before the age of five compared to children in high-income countries [1]. From the latest under-five statistics of low- and high-income countries, there is evidence of health inequalities among children globally. However, the United Nations 2030 Agenda’s Mandate for Sustainable Development argues towards combating health inequalities within and among countries [8].

Most countries globally have shown continuous effort in engaging health reforms leaning towards Universal Health Coverage (UHC) either by extending, deepening, or otherwise improving coverage with needed health services and financial protection [9]. Monitoring health inequalities is crucial in advancing health equity. The World Health Organization (WHO) priorities health equity hence the conceptualization of equity-oriented policies, such as the Universal Health Coverage policy. Immunization, food insecurity and malnutrition in children are timely issues relevant to the Global Nutrition Targets of 2025 and Sustainable Development Goals (SDGs), aiming to eliminate poverty and achieve zero hunger, good health, and well-being for all.

This article approaches these issues by comparing two countries in the BRICS economy block: South Africa and India. Brazil, Russia, India, China, and South Africa (BRICS) have been argued to be leading emerging economies and political powers at the regional and international level [1]. The BRIC acronym was initially coined in 2001, with the sole mandate of highlighting the unique role of important emerging economies with only Brazil, Russia, India, and China (BRIC). It was then that the four countries started to meet as a group in 2006, and South Africa was invited to join the group in 2010, which then transformed the acronym to BRICS [1,2]. Due to their geographic and demographic dimensions, BRICS economies severely influence global development, especially in Low-Income Countries (LIC). BRICS countries account for an estimated 25 per cent of the earth’s livable surface altogether, with about forty percent of the world’s total population [10,11]. The BRICS block has been argued to be a contender to challenge the developed economies for dominance in global economics, politics, and governance [12].

South African and India are part of the BRICS bloc. However, according to the human development index, India and South Africa are considered to have medium human development [13], with both having child food insecurity and malnutrition as substantial public health problems. As of 2014, 64% of children under six years of age lived in the poorest 40% of households [14], while the prevalence of stunting among preschool children was around 20% in 2015 [15], in South Africa. Similar statistics were reported in India, with malnutrition in children under five accounting for almost 50% of the 1.3 million annual deaths [16].

South Africa is a multicultural and multi-ethnic country, with some population groups still transforming from traditional-rural to urban lifestyles. The transition has been accompanied by a nutrition transition, which has been characterized by changes in dietary patterns and nutrient intakes [17]. At the same time, India is reported to be home to the largest number of hungry people in the world. India ranked 67/122 countries on the Global Hunger Index (GHI) in 2010 and 65/79 countries in 2012 on the same index [18]. As per a Global Survey Report of 2012, India ranked 112/141 countries on the child development index (CDI), and had disparities across various societies and states [19].

Health inequalities among under-five children were reported to have grown worse in South Africa [4], despite a reported increase in social spending on welfare, health, education and housing. South Africa’s child immunisation coverage exceeded the 2015 Global Vaccine Action Plan (GVAP) target; however, child health inequalities were cited, with more than one-quarter of their target population falling below 80% immunisation coverage in 2016 [3]. A study done in India showed that children with highly educated rich mothers aged 20–49 years belonging to the wealthiest 20% of the population are 5.3 times more likely to be vaccinated, compared to children born to teenage mothers with no education, from the poorest 20% of the population [8].

Stunting in South African children has been fluctuating high in the recent two decades, with significant declines recorded in; 1994; 22.9%, 1999; 21.6% and 2012; 21.5% [20]. In 2015, the World Bank argued that South Africa encounters a double burden of malnutrition, as undernutrition is prevalent among underweight and obese children [21]. 

In India, households with at least one stunted or wasted child were significantly poorer than other households in almost every dimension [22]. Malnutrition was reported to be more severe in rural than in urban India, with 50% rural children stunted and 21% wasted, while corresponding urban fractions were 40% stunted and 17% wasted [22].

Even though Sub-Saharan countries account for the highest prevalence’s of food insecurity, arguably in absolute terms, India has the highest number (25%) of food-insecure children [13,15,20]. A study done among 6–36-month-old children found more than 38% of children food insecure and child food insecurity as a predictor of malnutrition in India [23]. Malnutrition in children has been associated with long-term costs, for instance, adverse health conditions with consequent effects on labour [24]. However, in low-middle income countries, malnutrition in children poses a significant threat to poverty reduction [25,26].

Despite vast initiatives undertaken in recent decades, infant and child mortality fluctuates high globally, especially in developing countries [27], including South Africa and India. This study focused on socioeconomic inequalities in child food insecurity, child malnutrition and immunization coverages for the six childhood immunisable diseases (diphtheria, tetanus, pertussis, polio, measles, and tuberculosis) by comparing the two medium human development countries in the BRICS bloc; South Africa and India. The six childhood diseases account for the biggest proportion of deaths and constant sickness among children less than 12 months, and the latter diseases are also highly transmitted [28]. However, most studies reviewed in the literature examining child inequalities do not decompose the health inequalities in these three crucial indicators of child health to understand what could be driving the inequalities [1,4,6,8,15,17,20,22,23,24,25,28,29,30,31,32,33,34,35,36,37,38,39].

The proportion of total expenditure on health as a percentage of gross domestic product (GDP) has been cited to be lower in each of the BRICS countries than the global average (9.94%), ranging from 4.69% in India to 8.8% in South Africa [40]. [13] In 2015, India reported the highest under-five mortality rates (47.7%) in the BRICS, almost five times higher than the Russian Federation (9.6%) [41]. In addition, India and South Africa’s health systems have been described as the two most dismal performers in the BRICS block [42]. The initial intention of this study was to assess child disparities in the BRICS block using the latest available DHS data sets; however, South Africa and India only had the latest comparable data sets. Additionally, looking at Table 1, the socioeconomic indicators of both countries are almost similar. Therefore, the study estimated child health disparities between the two countries and locates where interventions are required for policy decisions. As highlighted by the WHO Thirteenth Global Programme of Work (GPW13), identifying health inequalities and their drivers is essential for achieving health equity [2].

## 2. Materials and Methods

### 2.1. The Study Area (Population)

As of 2018, gross domestic product (GDP) in purchasing power parity (PPP) terms were USD 11.326 trillion and USD 813,100 billion for India and South Africa, respectively, while their GDPs’ per capita was USD 8,378 and USD 13,865 for India and South Africa, respectively [43]. However, the reported Gini coefficients and Human Development Indices (HDI) of 2018 highlighted similar trends of income inequalities and standards of living; India: Gini (33.9%), HDI (0.640) and South Africa: Gini (63.0%), HDI (0.699) [43].

### 2.2. Data Sources

This study is based on South Africa’s 2016 and India’s 2015 Demographic and Health Survey (DHS). The primary objective of the 2015–2016 India demographic health survey was to provide essential data on health and family welfare, as well as data on emerging issues in health [44], while South Africa’s DHS objectives were to provide estimates of health and behaviour indicators among children aged 6–59 months and adults aged 15, and older [45]. South Africa’s 2016 demographic health survey used the Statistics South Africa Master Sample Frame (MSF), which was created using Census 2011 enumeration areas (EAs). In the MSF, EAs of manageable size were treated as primary sampling units (PSUs), whereas small neighboring EAs were pooled together to form new PSUs, and large EAs were split into conceptual PSUs [45].

While India’s demographic health survey sample of 2015 sample was a stratified two-stage sample [44], India’s 2015 DHS used the 2011 census as the sampling frame for selecting PSUs, with PSUs cited as villages in rural areas and Census Enumeration Blocks (CEBs) in urban areas. However, PSUs with fewer than 40 households were linked to the nearest PSU and within each rural stratum, villages were selected from the sampling frame with probability proportional to size (PPS).

After cleaning the data, study samples for children in India for Immunization (14,967) 0 to 9 months and Food Security and Nutrition 0 to 59 months (21,747), and for children in South Africa, were: Immunization (1062) 0 to 12 months and Food Security and Nutrition (378) 0–59 months. For this study, DHS study weights were recomputed by dividing women or children study weight variable (v005) by 1,000,000 [44]. Clustering of primary sample units was considered by using the PSU function in Stata.

### 2.3. Outcome Variables for Child Health

#### 2.3.1. Immunization: Fully Immunized

In this study, a child was considered fully immunized if; they had received one dose of Bacillus Calmette-Guerin (BCG), four doses of Oral Polio Vaccine (OPV), three doses of Pentavalent, three doses of Pneumococcal Vaccine (PCV), two doses of Rotarix (Rota) and one dose each of Measles and Yellow fever vaccines. A child is expected to receive a total of 7 vaccines and 15 doses [27,46]. A child was considered to have received the vaccine if the vaccination date was marked on the card. To avoid recall bias, child vaccinations by word of mouth from the mother without written confirmation on the child’s primary health care facility card were not considered immunized. In this study, the child immunization age was limited to 9 months [27,46]. 

#### 2.3.2. Food (In)Security

Children’s food security was determined using the WHO dietary diversity scores approach, the Infant and Young Child Feeding (IYCF) practices. Dietary diversity is defined as the number of different foods or food groups consumed over a given reference period [47]. In this study, we considered 13 food groups. The 13 food groups were food from grains, food from tubers, eggs, meat, pumpkin and carrots, green leafy vegetables, vitamin A fruits, other fruits, liver & heart, fish, (beans, peas, lentils, nuts), other milk products and yoghurt. The study adopted the Infant and Young Child Feeding (IYCF) minimum dietary diversity indicator for food security. The minimum dietary diversity indicator has a cut-off point of greater than four food groups (>4) [47]. Therefore, in this study, children with at least 3 of the 13 food groups were defined as food insecure.

#### 2.3.3. Malnutrition

The study adopted a child anthropometric measure of weight-for-age for assessing malnutrition. Weight-for-age is a composite index of height-for-age and weight-for-height that considers both acute and chronic under-nutrition [48]. Children whose weight-for-age z-score was below minus two standard deviations (−2 SD) from the median were considered malnourished.

### 2.4. Methods

#### 2.4.1. Socioeconomic Status: Wealth Indicator

The study used household wealth indices based on household assets; the wealth indices were used as a proxy indicator of the households’ Socioeconomic Status (SES). It was constructed by assigning household scores, then each person was ranked in the household population by their score, and lastly, the distribution was divided into five equal categories, each with 20% of the population in the original studies with economic proxies, such as housing quality, household amenities, consumer durables and size of landholding [44,45,49]. The household wealth indices used in this study were adopted from Demographic Health Surveys of 2015/16 for India and South Africa. Socioeconomic status was adopted as reported by the DHS 5 groups (poorer, poor, middle, richer, richest).

#### 2.4.2. Statistical Analysis

##### Socioeconomic Inequality in Child Health Outcomes

The concentration index (CI) is a standard method used in assessing health inequalities [50]. The CI is twice the area between the concentration curve and the line of equality [51], calculated as follows:

(1) $$CI=\frac{2}{n\mu }\overset{n}{\underset{i}{\sum }}=1Health_{i}R_{i}-1$$

where: *Health* is the health status of the ith individual; *µ* is the mean of the health variable; *R_i_* is the fractional rank of the ith individual in the income distribution; Erreygers Normalized concentration index

Concentration indices tend to misestimate the extent of health inequalities when the health variable is a bounded variable [52,53]. Since child immunization, child food security and child nutrition are bounded variables, the study adopted the Erreygers Normalized concentration index (ENCI). The Erreygers Normalized concentration index is expressed as follows:

(2) $$ENCI=f\left(\mu_{n},n\right)\overset{n}{\underset{i}{\sum }}=1Z_{i}h_{i}$$

where: *Z_i_* represents the number of individuals in each population; *I* denote the socioeconomic rank of the individual ranging from the richest to the poorest; *h* represents the health situation of the whole population

The Erreygers Normalized concentration index (ENCI) value ranges from −1 to 1, and the larger the absolute value of the ENCI the more severe the health inequalities [54]. When the ENCI is 0, health endowments will be equally distributed between poor and rich children. When the ENCI is positive, health endowments are concentrated among the rich children; hence, pro-rich health inequalities exist. When the ENCI is negative, health endowments are concentrated among the poor, thus the existence of pro-poor health inequalities.

##### Decomposition of the Erreygers Normalized Concentration Index

The Erreygers Normalized concentration index can be decomposed into the contributions of explanatory factors using regression analysis, thus enabling analysis of each determinant’s contribution to the extent of socioeconomic inequalities in health [55,56]. Income-related health inequalities were decomposed into the contributions of various explanatory factors, with each contribution as the product of the elasticity of health. Assuming a linear relationship between our child health outcomes (*yi*) and a set of k explanatory variables will be:

(3) $$y_{i}=a+\underset{k}{\sum }\beta_{k}X_{ki}+\varepsilon_{i}$$

Wagstaff et al. showed that for any health variables exhibiting a linear relationship with a set of k exploratory variables, the concentration index for the health variable could be decomposed as follows:

(4) $$\;CI=\sum_{k}\left(\frac{\beta_{k}\;{ẋ}_{k}}{\hat{y}}\right)CI_{k}+\frac{GCI_{\epsilon }}{\hat{y}}$$

where: *β_k_* is the partial; *ŷ* is the mean of the health variable; *ẋ_k_* is the mean of *ẋ_k_*; *CI_k_* denotes the concentration index of *x_k_* against income; *GC_ɛ_* is the generalized concentration for the error term.

Equation (4) can be modified as shown below to decompose the Erreygers concentration index [57]:

(5) $$E_{c}=4\left[\sum_{k}\left(\beta_{k}\;{ẋ}_{k}\right)CI_{k}+GCI_{\varepsilon }\right]$$

##### Concentration Curves

Concentration curves represent how immunization coverage, food insecurity, and malnutrition in children are distributed across subgroups with an inherent ordering of socioeconomic status. Thus, the concentration curve plotted the cumulative share of immunization coverage, food insecurity and malnutrition against the cumulative share of children ranked from poorest to richest.

If perfect health equality exists, the concentration curve will be a 45-degree diagonal line (line of equality); thus, the share of immunization coverage, food insecurity and malnutrition will exactly match the share of children belonging to each wealth rank. When the health inequalities are dominant among the poor, the concentration curve will lie below the line of equality, and if dominant among the rich, the curve will lie above the line of equality. The further the concentration curve lies from the equality line, the greater the health inequality [58].

## 3. Results

The overall prevalence of child full immunization, food insecurity and malnutrition in India was 66.11, 79.78 and 39.34%, respectively, while South Africa reported 33.41, 85.35 and 7.87%. India’s full child immunization was dominant among children belonging to the three lower socioeconomic quintiles (poorest; 20.11%, poorer; 22.23% & middle; 20.90%) [Table 2]. Children residing in rural India (71.54%), whose parents had secondary education (mother’s education; 52.21%; father’s education; 57.72%), had gone for more than four antenatal care visits (56.83), delivered at a healthy facility (89.26%), the child being the first (first birth order; 40.65%) and mother’s with no media exposure (62.53%) accounted for the biggest proportions of fully immunized children (Table 2).

In South Africa, full child immunization was dominant among children belonging to the three lower socioeconomic quintiles (poorest; 22.04%, poorer; 22.51% & middle; 22.14%) (Table 2). Furthermore, in South Africa, children residing in the urban areas (61.20%), whose mothers had secondary education (80.54%), belonged to female-headed households (52.44%), had gone for at least four antenatal care visits (81.71%) and mother’s with no media exposure (38.44%) accounted for the biggest proportion of fully immunized children [Table 2].

Child food insecurity and malnutrition were more prevalent among the three lowest socioeconomic groups (poorest, poorer & middle) in both countries (Table 3). However, in India, child food insecurity (74.76%) and malnutrition (78.38%) were more dominant among rural children, while in South Africa, child food insecurity (65.25%) and malnutrition (53.76%) were dominant among children in the urban areas (Table 2). For India, bigger households with more than five members accounted for the biggest proportions of food-insecure and malnourished children (Table 2).

### 3.1. Concentration Indices and Curves

India’s full immunization concentration index (0.1640) was positive (pro-rich), thus favoring children from wealthy households, while for South Africa it was negative (pro-poor) thus, children from poor households were fully immunized (Table 3). Food security concentration indices for both countries were negative, meaning poor children in both countries were food secure (Table 3), while child nutrition also reported pro-poor concentration indices for both countries (Table 3). Therefore, in both countries, poor children had adequate nutrition than children from wealthy households. However, only India’s concentration indices on child immunization, food security and nutrition were statistically significant at 95% confidence interval (Table 3).

**Figure 1:**
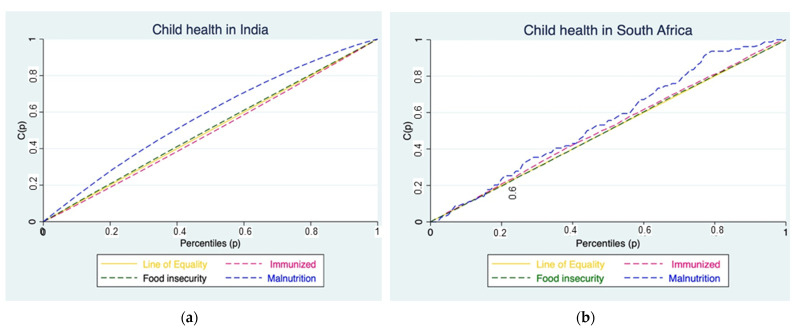
Concentration curves of Immunisation, Food security and Nutrition for (**a**) India & (**b**) South Africa.

Figure 1a,b gives a graphical presentation of the indices presented in Table 3. However, some of the curves in South Africa crossed the line of equality which prompted the computation of the dominance tests (Table 4). Test of dominance for the India analysis between food security concentration curve and 45^0^ lines showed that the food security concentration curve dominates (Table 4). At the same time, a test of dominance for the South Africa analysis between the fully immunized concentration curve and 45^lines^ne showed that the fully immunized concentration curve dominates (Table 4). Test of dominance between food security concentration curve and 4linesine for South Africa showed non-dominance (Table 4). Finally, the test of dominance between the nutrition concentration curve and the line of equality showed that the nutrition concentration curve dominates (Table 4).

Since the curves crossed the line of equality at some points, we computed the dominance test, and all curves were non-dominant, meaning that the concentration curves dominated thlines line.

### 3.2. Decomposition Results

#### 3.2.1. Child Immunization

Major positive contributors of health inequalities in child immunization for India were household wealth (30.73%), mother’s education (27.08%), place of delivery (14.63%) and antenatal care (16.83%) (Table 5). However, South Africa had both negative and positive significant drivers of socioeconomic health inequalities in child immunization. Major negative drivers of inequalities in immunization were household wealth (−4.3 × 10^2^%) and mother’s education (−1.2 × 10^2^%), while residence status (174.64%), mother’s age (231.06%), media exposure (81.53%) and birth order (109.13%) were positive drivers (Table 6).

#### 3.2.2. Food Insecurity

With regards to socioeconomic inequalities on child’s food insecurity in India, mother’s education (108.20%) (Table 5), was the major positive driver while for South Africa, household wealth was the major positive drive (336.80%) and husband’s education (−1.8 × 10^2^) negatively drove food insecurity health inequalities (Table 6). 

#### 3.2.3. Malnutrition

Socioeconomic inequalities in child malnutrition in India were mainly driven by household wealth (69.00%) and mother’s education (24.56%) (Table 5). In South Africa, household wealth (31.90%), mother’s education (20.23%), husband’s education (22.51%) and mother’s age (20.31%) contributed positively to the child malnutrition socioeconomic inequalities (Table 6).

## 4. Discussion

The study assessed socioeconomic inequalities in child health using the following indicators: immunization among children aged between 0–9 months, food insecurity and malnutrition among children aged 0–59 months in India and South Africa. Pediatric immunization has been attributed as one of the crucial health interventions in modern times [59]. Evidence in literature has shown substantial reductions in child mortality globally due to pediatric immunization [60].

Studies done in India and other countries recently reported findings that argued that higher maternal education induced utilization of health care services, and one of the increased health service uptakes were increased vaccine uptake among children [5,36]. The latter was also corroborated in our study results, as more child immunization, less food insecurity and malnutrition were reported among children with educated parents. A previous study was done in India, using rounds of the National Family Health Survey (NFHS), reported low immunization coverage among children in rural compared to urban locations [61]. Pro-rich inequalities have characterized Low-middle income countries in child immunization [6,31,34]. Our findings concur with other studies done in India, Nigeria, and Pakistan, where partial or never immunized children were dominant among the poor [30,39,59].

Decomposed results on child immunization showed household wealth, residence status, household head sex, antenatal care, postnatal care, place of delivery and mother’s education as major drivers of health inequalities in child full immunization for India and South Africa. The latter results are in harmony with findings of a study done in India, which showed the same child immunization determinants as main drivers of child immunization socioeconomic inequalities contributing about 97% of total socioeconomic inequalities [30].

Our study also reported high food insecurity prevalence among poor rural nourished children whose mothers had attained at least secondary education for both countries. The observed disproportionate health inequalities reported in India and argue that, even though children were receiving nutritious food, challenges in food access were being experienced; this was also observed in other studies [35,58,62,63]. Computed concentration curves for food security status in India and South Africa were all negative, indicating that children from wealthy households were food secure, concurring with findings in the literature [7,22,29,32,37,58,62,63,64,65,66]. The differences between quantity and quality dietary intake among children have been widely documented in literature [7,64,65,66,67]. Our study observed similar findings in both countries as food-insecure children accounted for the biggest proportions of malnourished children. However, it was only India’s results on food insecurity and malnutrition that confirmed the generalized hypothesized argument in literature, that food insecurity and malnutrition are more dominant in the peri-urban and rural locations [7,15,16,17,22,23,24,29,32,34,35,37,58,62,63,65,66,67,68,69].

Decomposed concentration indices of food security reported child age, nutrition, household wealth, place of residence, and mother’s and partner’s education as major drivers of child health inequalities in food security in India. The child food security determinants have also been argued to be drivers of child health inequalities in other studies [22,29,35,38,62,68,69,70,71].

However, for South Africa and household wealth and other mentioned determinants, mother’s age was a major driver of health inequalities in food security, consistent with other study findings observed in the literature [63,72,73,74,75,76]. The decomposed results also reported household wealth as the main driver of child health inequalities in food security in both countries, reflecting possible similar income inequalities between India and South Africa. In India, residence status was a positive contributor to child health inequalities relative to food security, while in South Africa, residence status was a negative contributor. Thus, where the child stayed in India would widen the inequalities, and for South Africa, it would reduce the inequalities. Therefore, the negative contribution to child health inequalities of residence status in South Africa relative to food security confirms arguments posed in some studies in the literature of the disappearance of so-called urban advantages [77].

Lack of education has been argued to undermine productivity, employability and earning capacity, leading directly to poverty and hunger [78]. In this study, maternal education in India widened child health inequalities relative to food security, while in South Africa, it reduced the inequalities. The widening of child health inequalities in India due to maternal education can be attributed to complex social and cultural beliefs in many developing countries [19]. However, in South Africa, it can be deduced that mothers are fully aware that children should eat. We observed quite a unique finding when child health inequalities were decomposed relative to nutrition. The study observed that maternal education widened health inequalities relative to child nutrition. This means that even though mothers understood the previous messages that children should eat to avoid kwashiorkor, mothers have focused more on quantity (food security) with no regard for quality (nutrition).

Household head sex reduced child health inequalities relative to food security in both countries, which is astounding as South Africa is characterized by relatively more female-headed households than India. However, household head sex widened child nutrition inequalities in South Africa and reduced the inequalities in India. Therefore, this means that even though the female-headed households in South Africa are fully aware that children should eat, there is still a gap in knowledge on the quality of food to be given to children. In both countries, the decomposed results showed that household size widened the child health inequalities relative to food security; however, relative to nutrition, household size reduced food availability for consumption in South Africa, while in India, it was the opposite, although India has a larger average household size compared to South Africa.

Decomposed concentration indices of nutrition across child health determinants in India showed household wealth, child age, mother’s, and partner’s education as major drivers of child health inequalities in nutrition. These results concurred with findings from other studies [24,79,80,81,82,83]. However, for South Africa, apart from socioeconomic status and mother’s education, household head sex, household size, mother’s age, mother’s, and partner’s education were significant drivers of child health inequalities in nutrition, consistent with what other studies observed [22,29,35,38,62,68,69,70,71]

Achieving Universal Health Coverage, countries would have positively contributed to at least 6 of the 17 Sustainable Development Goals. Attainment of Universal Health Coverage implies; financial risk protection, access to quality essential healthcare services and access to safe, effective, quality and affordable essential medicines and vaccines for all [84]. Without a doubt, immunization is the only intervention in health systems that brings most households into contact with the health systems five or more times during the first year of a child’s life [85]. Therefore, high immunization coverage among children can be used as leverage in providing essential services, thus eventually increasing the financial efficiency of health systems. However, as reflected in our study, the socioeconomic gradient exists in India, posing a threat to the mantra of not leaving a child behind.

The major global impacts of food insecurity and malnutrition have long been recognized. Poor diets are argued to be among the leading causes of health and societal challenges, which eventually leads to disability and death, driving health inequalities and staggering healthcare costs in the 21st century [2,17,24,29,35,55,56,63,64,65,67]. Our study showed how crucial understanding and integrating child food insecurity and malnutrition in the Universal Health Coverage framework is, considering the observed socioeconomic inequalities in child health for India and South Africa.

If particular attention is not paid to the widening socioeconomic inequalities in food insecurity and malnutrition in developing countries [7,27,29,32,34,35,37,38,62,63,69,86], it will be impossible not to leave children behind.

## 5. Policy Recommendations

Immunization of children can be used as an entry point to Universal Health Coverage, as the intervention is considered one of the most cost-effective interventions [87]. Our results provide evidence which argues for health intervention strategies to reduce socioeconomic inequalities in immunization for India, supplemented with strategies that also target poverty reduction and erosion of illiteracy among women.

It is next to impossible to attain Universal Health Coverage without availing access to quality nutrition services for all. Literature has reported the cost of addressing malnutrition and nutrition-related diseases to be huge and significant, with reported associated losses to the economy estimated to be about USS 3.5 trillion annually [70]. A strong interdependence between nutrition security and social protection has been argued to exist in the literature [88]. Therefore, ensuring social protection can help in addressing the underlying critical determinants of malnutrition.

Therefore, it makes economic sense for governments and partners to make policy and financial commitments that fully integrate nutrition interventions into national health systems to achieve quality Universal Health Coverage. It is essential to make nutrition services part of the standard package of available healthcare services and universally available to vulnerable populations with higher levels of undernutrition, morbidity, and mortality rates [71].

Village or community health workers are an essential tool in addressing socioeconomic inequalities in malnutrition in developing nations. For instance, in Mali, the Action Against Hunger’s project with the Innocent Foundation expanded community health workers’ package of interventions to include diagnosis of malnutrition and treatment [89]. Due to the latter initiative, promising results have been observed, with current results reporting that 95% of malnourished children treated by community health workers in the community recovered, compared with 88% of those treated at health facilities [89]. This is among many of the strategies that can be used in tackling child health inequalities.

## 6. Study Limitations

The key assumption of this study is that the regression modelling done when decomposing the concentration indices is independent of the predictors. We understand that the social variables included in the study may be correlated or causal related this is the limitation of this study methodology. However, several studies in the literature have used similar child health determinants as determinants in their regression models [4,5,27,36,59].Additionally, the initial intention of the study was to assess child health inequalities in the BRICS block using the DHS data sets. However, only India and South Africa had comparable data sets, with some countries missing or outdating data.

## 7. Conclusions

There are socioeconomic inequalities in child immunization, food insecurity and malnutrition in India and South Africa. The main drivers of the socioeconomic inequalities in both countries across the child health outcomes (immunization, food insecurity and malnutrition) are household wealth, mother’s age, child age, household size, mother’s education, and partner’s education. In conclusion, if growing socioeconomic inequalities in children are not addressed in developing countries, “some children will definitely be left behind”.

## Figures and Tables

**Table 1:** Socioeconomic indicators in the BRICS block as of 2017.

	India	South Africa	Brazil	China	Russia
Crude birth rate (per 1000 persons)	20.4	21.3	13.6	12.4	11.5
Crude death rate (per 1000 persons)	6.4	9.0	6.2	7.1	12.4
Infant mortality rate (per 1000 live births)	34.0	32.8	12.8	7.5	5.6
Public expenditure on health as % of GDP	1.2	4.2	4.9	6.2	3.1

Source: [40].

**Table 2:** Immunisation, food insecurity and malnutrition percentage prevalence across child health determinants in India and South Africa for 2015/16.

	India			South Africa		
Child Health Prevalence	Fully Immunised (%)	Food Insecure (%)	Malnutrition (%)	Fully Immunised (%)	Food Insecure (%)	Malnutrition (%)
Socioeconomic status						
Poorest	20.11 ***	27.14 ***	35.03 ***	22.04 *	21.09 **	24.93
Poorer	22.23 ***	23.11 ***	25.24 ***	22.51 *	23.71 **	30.39
Middle	20.90 ***	20.16 ***	18.93 ***	22.14 *	22.39 **	21.21
Richer	19.49 ***	16.76 ***	13.40 ***	19.73 *	16.92 **	17.58
Richest	17.27 ***	12.84 ***	7.40 ***	13.57 *	15.88 **	5.89
Residence status						
Urban	28.46 ***	25.24 ***	21.62 ***	61.20 ***	65.25	53.76
Rural	71.54 ***	74.76 ***	78.38 ***	38.80 ***	34.75	46.24
Mothers education						
No education	21.64 ***	31.21 ***	38.41 ***	1.41 **	1.78	5.24 ***
Primary	12.04 ***	14.45 ***	15.81 ***	6.90 **	9.38	6.99 ***
Secondary	52.21 ***	44.75 ***	40.33 ***	80.54 **	76.46	84.32 ***
Tertiary	14.11 ***	9.59 ***	5.46 ***	11.15 **	12.37	3.45 ***
Husband’s education						
No education	12.87 ***	17.81 ***	23.25 ***	3.27	4.16	11.68 **
Primary	12.28 ***	14.57 ***	17.24 ***	9.76	11.12	0.00 **
Secondary	57.72 ***	54.60 ***	51.30 ***	69.86	69.69	86.40 **
Tertiary	17.13 ***	13.03 ***	8.21 ***	17.12	15.03	1.92 **
Household head sex						
Male	87.33	87.73	87.36	47.56 ***	57.13	55.25 *
Female	12.67	12.27	12.64	52.44 ***	42.87	44.75 *
Antenatal care						
<4ANC visits	43.17 ***			18.29 **		
>4ANC visits	56.83 ***			81.71 **		
Postnatal care						
No PNC	58.92 ***			17.72		
Attended PNC	41.08 ***			82.28		
Place of delivery						
Home	10.74 ***			3.40		
Health facility	89.26 ***			96.60		
Birth order						
1st	40.65 ***			36.43		
2nd	34.51 ***			31.54		
3rd	14.25 ***			18.92		
4^+^	10.59 ***			13.11		
Media exposure						
No media exposure	62.53 ***			38.44 *		
Less than once a week	15.95 ***			24.18 *		
Almost daily	21.52 ***			37.38 *		
Nutrition status						
Nourished		61.13 **			92.27	
Malnourished		38.87 **			7.73	
Child sex						
Male		49.72	48.75 *		52.48	51.74
Female		50.28	51.25 *		47.52	48.26
Food security status						
Food secure			21.16 **			18.32
Food insecure			78.84 **			81.68
Household size						
5/3 Members		38.55 ***	38.90 **		30.79	9.84
5^+^/3^+^ Members		61.45 ***	61.10 **		69.21	90.16

Percentage. [*** *p* < 0.01, ** *p* < 0.05, * *p* < 0.1] Statistical significance of the Chi-square Test. Note: Average household size in India is 5 while in South Africa it is 3.

**Table 3:** Erreygers normalised concentration indices for child health indicators for South Africa and India.

India		
Erreygers Normalised Concentration Index		
Immunisation	0.1640 *** (0.0056)	(Note: Standard error adjusted for 26037 clusters in primary sampling unit)
Food insecurity	−0.0548 *** (0.0059)	
Malnutrition	−0.2314 *** (0.0048)	(Note: Standard error adjusted for 25661 clusters in primary sampling unit)
**South Africa**		
**Erreygers Normalised Concentration Index**		
Immunisation	−0.0236 (0.0226)	(Note: Standard error adjusted for 679 clusters in primary sampling unit)
Food insecurity	−0.0117 (0.0220)	
Malnutrition	−0.0351 (0.0181)	(Note: Standard error adjusted for 542 clusters in primary sampling unit)

Note: (Robust Standard Error); *** *p* < 0.01.

**Table 4:** Test of dominance between child health indicators concentration curves and 45-degree line (line of equality) for South Africa.

Variable	Significance Level	Number Points	Rule
Immunisation	5%	19	mca
Food security	5%	19	mca
Malnutrition	5%	19	mca
Non-dominance			

**Table 5:** Decomposition child health indicators across child health determinants in India.

	Immunisation				Food Security				Nutrition			
	Elasticity	Concentration Index	Contribution	Contribution (%)	Elasticity	Concentration Index	Contribution	Contribution (%)	Elasticity	Concentration Index	Contribution	Contribution (%)
Food insecurity									−0.0252	−0.0172	0.0017	−0.7480
Malnutrition					−0.0093	−0.1470	0.0055	−9.9556				
Household wealth	0.0439	0.2870	0.0504	30.7361	−0.0069	0.2870	−0.0079	14.4277	−0.1391	0.2870	−0.1597	69.0023
Residence status	0.0061	−0.0697	−0.0017	−1.0411	0.0314	−0.0697	−0.0088	15.9989	−0.0230	−0.0697	0.0064	−2.7763
Mothers education	0.0462	0.2405	0.0444	27.0784	−0.0616	0.2405	−0.0593	108.2039	−0.0591	0.2405	−0.0568	24.5562
Husbands education	−0.0041	0.1688	−0.0028	−1.6769	0.0172	0.1688	0.0116	−21.2282	−0.0186	0.1688	−0.0126	5.4366
Mothers age	0.0923	−0.0043	−0.0016	−0.9585	−0.0559	−0.0043	0.0010	−1.7370	−0.0299	−0.0043	0.0005	−0.2202
Household head sex	0.0097	−0.0087	−0.0003	−0.2046	−0.0513	−0.0398	0.0082	−14.8992	0.1050	−0.0398	−0.0167	7.2264
Child sex					0.0205	−0.0087	−0.0007	1.2982	−0.0099	−0.0087	0.0003	−0.1478
Household size					0.0001	−0.0098	−0.0000	0.0085	−0.0058	−0.0098	0.0002	−0.0972
Media exposure	−0.0021	0.4366	−0.0037	−2.2752								
Antenatal cares	0.0353	0.1957	0.0276	16.8309								
Postnatal care	0.0223	0.0536	0.0048	2.9094								
Delivery place	0.0787	0.0762	0.0240	14.6296								
Birth order	−0.0226	−0.1719	0.0155	9.4771								
Residuals	4.50%				7.88%				−2.23%			

**Table 6:** Decomposition of child health indicators across child health determinants in South Africa.

	Immunisation				Food security				Nutrition			
	Elasticity	Concentration Index	Contribution	Contribution (%)	Elasticity	Concentration Index	Contribution	Contribution (%)	Elasticity	Concentration Index	Contribution	Contribution (%)
Food insecurity									0.0206	−0.0034	−0.0003	0.8050
Malnutrition					0.0041	−0.1114	−0.0018	15.5031				
Household wealth	0.1147	0.2716	0.1247	−4.3 × 10^2^	−0.0516	0.2716	−0.0561	336.7970	−0.0103	0.2716	−0.0112	31.8986
Residence status	0.1073	−0.0962	−0.0413	174.6443	−0.0096	−0.0962	0.0037	−31.5812	−0.0078	−0.0962	0.0030	−8.5374
Mothers education	0.1264	0.0568	0.0287	−1.2 × 10^2^	0.0109	0.0568	0.0025	21.1586	−0.0312	0.0568	−0.0071	20.2332
Husbands education	−0.0149	0.0736	−0.0044	18.5961	0.0699	0.0736	0.0206	−1.8 × 10^2^	−0.0268	0.0736	−0.0079	22.5091
Mothers age	−0.6896	0.0198	−0.0546	231.0637	0.0891	0.0198	0.0071	−60.2722	0.0899	0.0198	0.0071	20.3118
Household head sex	0.0354	−0.0213	−0.0030	12.7870	0.1697	0.0026	0.0017	−14.9082	−0.0318	0.0026	−0.0003	0.9317
Child sex					−0.0109	−0.0213	0.0009	7.9502	−0.0525	−0.0213	0.0045	12.7762
Household size					−0.0057	0.0045	−0.0001	0.8688	0.0199	0.0045	0.0004	1.0195
Media exposure	−0.0248	0.1943	−0.0193	81.5340								
Antenatal cares	0.0298	0.0275	0.0033	−13.8813								
Postnatal care	0.0609	0.0122	0.0030	−12.5641								
Delivery place	−0.1957	0.0133	−0.0104	44.0679								
Birth order	0.1006	−0.0641	−0.0258	109.1246								
Residuals	4.63%				4.48%				−1.95%			

## Data Availability

DHS is a secondary data analysis hence data sets are publicly available on https://dhsprogram.com/data/available-datasets.cfm (accessed on 12 October 2019).
